# 人肺腺癌脑转移动物模型建立及显像的研究

**DOI:** 10.3779/j.issn.1009-3419.2013.08.01

**Published:** 2013-08-20

**Authors:** 贝 雷, 杰 曹, 杰 沈, 兰香 赵, 胜 梁, 庆刚 孟, 文晖 谢, 顺芳 杨

**Affiliations:** 1 200030 上海，上海交通大学附属胸科医院核医学科 Department of Nuclear Medicine, Shanghai Chest Hospital, Shanghai Jiao Tong University, Shanghai 200030, China; 2 200030 上海，上海交通大学附属胸科医院病理科 Department of Radiology, Shanghai Public Health Clinical Centre of Fudan University, Shanghai 201508, China; 3 201508 上海，复旦大学 附属上海市公共卫生中心放射科 Department of Pathology, Shanghai Chest Hospital, Shanghai Jiao Tong University, Shanghai 200030 China; 4 200025 上海，上海交通大学附属瑞金医院核医学科 Department of Nuclear Medicine, Shanghai Ruijin Hospital, Shanghai Jiao Tong University, Shanghai 200025, China; 5 201210 上海，复旦大学药学院 Department of Pharmaceutics, School of Pharmacy, Fudan University, Shanghai 201210, China

**Keywords:** 肺肿瘤, 肿瘤转移, 脑, 动物模型, PET, MRI, Lung neoplasms, Tumor metastasis, Brain, Animal model, Positron emission tomography (PET), Magnetic resonance imaging (MRI)

## Abstract

**背景与目的:**

肺癌脑转移是临床常见的严重并发症，由于脑部结构和功能的特殊性、脑转移检测方法的局限性，预后很差。本研究旨在筛选人肺腺癌脑转移细胞株CPA-Yang1-BR以及建立裸小鼠动物模型和检测方法。

**方法:**

将人肺腺癌细胞株CPA-Yang1-GFP接种于裸小鼠左心室，约7周-8周后比较三种小动物显像方法：micro PET/CT显像，X线、放射性核素、荧光（三合一）活体成像系统和小动物线圈MRI显像，实验证明MRI显像是最准确的小鼠脑转移病灶检测方法。脑核磁共振成像（magnatic resonance imaging, MRI）显像找到脑转移灶，深麻醉处死裸小鼠取出脑转移病灶，部分病理验证，部分行原代培养后获得人肺腺癌脑转移细胞，再次接种裸小鼠，用上述方法重复以上体内外循环4次，观察脑转移形成情况。

**结果:**

获得人肺腺癌脑转移细胞株CPA-Yang1-BR及其裸小鼠模型。

**结论:**

CPA-Yang1细胞经反复裸小鼠脑组织内外筛选的方法可获得具有高转移潜能的裸小鼠脑转移模型，为肺癌脑转移的生物学研究提供了一个良好的技术平台。小动物线圈MRI或micro MRI活体显像是检测小鼠肺癌脑转移敏感、准确、无创伤的显像方法。

## Abstract (en)

**Background and objective:**

Lung cancer is the leading cause of cancer-related death in men and women. It is also the most common cause of brain metastases. A brain metastasis model is difcult to be established because of the presence of the blood-brain barrier (BBB) and the lack of optimal methods for detecting brain metastasis in nude mice. Tus, the establishment of a Chinese lung adenocarcinoma cell line and its animal model with brain metastasis potency and *in vivo* research is of great signifcance.

**Methods:**

CPA-Yang1 cells were obtained from a patient with human lung adenocarcinoma by lentiviral vector-mediated transfection of green fluorescence protein. Intracardiac inoculation of the cells was performed in nude mice, and brain metastatic lesions were detected using micro ^18^F FDG-PET/CT scanners, small animal *in vivo* imaging system for fluorescence, radionuclide and X ray fused imaging, magnetic resonance imaging (MRI) with sense body detection, and resection. The samples were divided into two parts for cell culture and histological diagnosis. The process was repeated *in vivo* and *in vitro* for four cycles to obtain a novel cell clone, CPA-Yang1-BR.

**Results:**

A novel cell clone, CPA-Yang1-BR, was obtained with a brain metastatic rate of 50%. The use of MRI for the detection of brain metastases has obvious advantages.

**Conclusion:**

An experimental Chinese lung adenocarcinoma cell clone (CPA-Yang1-BR) and its animal model with brain metastasis potency in nude mice were established. MRI with sense body or micro MRI may be used as a sensitive, accurate, and noninvasive method to detect experimental brain metastases in intact live immunodefcient mice. The results of this study may serve as a technical platform for brain metastases from lung adenocarcinoma.

肺癌已连续多年是全世界癌症死亡的主要原因^[1]^，近年来也已成为我国发病率和死亡率第一位的恶性肿瘤^[2]^。临床上，肿瘤的转移很少仅限于某个单脏器转移，尤其肺癌的转移：骨(35%-80%)、肾上腺(53.7%)、肝(22%)、脑(10%)^[3-6]^。虽说脑转移所占比例不大，但是危害性极大，一旦患者出现脑转移预后极差。肺癌脑转移是肺癌患者终末期表现之一，生存期很短，未经治疗中位生存期仅1-3个月^[7]^。从肺癌组织脱落的肿瘤细胞不受肺毛细血管床的阻滞，可以直接进入体循环，流向脑部，因为有血脑屏障(blood-brain barrier, BBB)存在，能保护中枢神经系统免受癌细胞侵袭，即使部分癌细胞进入内皮细胞层，BBB也能积极参与阻止转移性细胞在大脑中溢出和扩散^[8]^。但是仍无法完全阻止部分癌细胞向脑组织侵袭，导致肺癌脑转移的发生。有20%-30%的肺癌患者在诊断时即已经发生了脑转移，40%-50%的肺癌患者在整个病程中会出现脑转移^[9, 10]^。但其具体转移机制仍然未知，研究方法也较少，使得临床治疗疗效很差，迫切需要进一步的研究。肺癌脑转移细胞及其实验动物模型的建立是研究肺癌脑转移的重要基础，但由于脑部的功能及结构的特殊性、脑转移检测方法的有限性，使肺癌脑转移模型的建立非常困难。CPAYang1是偶然发现产生骨转移同时伴发脑转移的人肺腺癌细胞株，一次接种约有22%的裸小鼠产生脑转移。借鉴以往应用裸小鼠体内反复接种可以筛选到某一器官特异性转移亚株的方法^[11-15]^，本研究应用慢病毒介导绿色荧光蛋白转染CPA-Yang1(green ﬂuorescence protein, GFP)细胞，经裸小鼠左心室接种，MRI显像和脑组织解剖，检出并取出脑转移灶行细胞原代培养及病理检测，体内外反复筛选获得较高潜能产生脑转移的细胞亚株CPA-Yang1-BR，一次接种可有1/2的裸小鼠产生脑转移。本研究改善了以往研究方法特异性差、成功率低、操作复杂等缺点，构建了人肺腺癌脑转移细胞及其动物模型，建立了检出脑转移灶的方法以及深入研究的实验平台。

## 1 材料与方法

### 1.1 实验动物

8周-10周龄、体重18 g-20 g的雄性BALB/c裸小鼠，由上海市西普尔-必凯实验动物有限公司提供，在SPF环境下饲养[实验动物使用许可证号：SYXK(沪) 2008-0043]。

### 1.2 肿瘤细胞

本课题组自建可传代中国人肺腺癌细胞株CPA-Yang1^[11]^(国家发明专利号：No.200810200983.8)，取自首诊伴发颞骨溶骨转移人肺腺癌患者的心包积液培养而成。细胞培养用10%胎牛血清的DMEM/F12培养基，置于37 ℃、CO_2_体积分数为5%的培养箱(Heraeus BB-16 CO_2_ Incubators, Germany)中培养。血清及培养液购自美国Gibco公司(GibcoBRL, Carlsbad, CA, USA)。慢病毒介导绿色荧光蛋白(GFP)转染CPA-Yang1细胞的实验由上海吉凯基因化学技术有限公司完成(图 1)。

**1:**
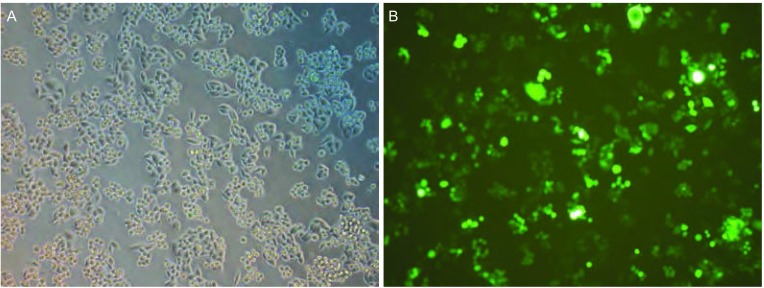
慢病毒介导转染GFP后的CPA-Yang1细胞。A：倒置显微镜光镜下的细胞；B：荧光倒置显微镜下的A图细胞（×100）。 CPA-Yang1 with lentiviral vector-mediated transfection of green fluorescence protein (GFP). A: light-field; B: green fuorescence (×100).

### 1.3 肺腺癌脑转移模型的建立

#### 1.3.1 左心室注射建模^[12, 13]^

取经体外培养的对数生长期CPA-Ya ng1 GF P细胞，每只裸小鼠左心室接种7×10^5^-8×10^5^/0.1 mL细胞悬液。左心室注射2周后隔天称重，体重低于16 g时严密观察有否出现嗜睡、行动迟缓等症状或异样体征。

#### 1.3.2 动物影像学检测

左心室接种造模7周-8周，当观察到裸小鼠全身严重衰竭、呼吸短促、体重减轻20%时，为裸小鼠腹腔注射硫喷妥钠麻醉后行micro PET/CT显像、X线、放射性核素、荧光(三合一)活体成像系统和小线圈MRI显像。

##### 1.3.2.1 Micro PET/CT scanners显像(Inveon micro PET/CT, Siemens Preclinical Solution, Knoxville, TN, USA)

对荷CPA-Yang1细胞的裸鼠尾静脉注射^18^F-FDG 0.2 mci/只，30 min后行micro CT和micro PET显像。

##### 1.3.2.2 三合一活体显像系统[Kodak FX PRO in vivo Imaging System Multispectral (Kodak, USA)]

显像前2 h-3 h每只小鼠尾静脉注射放射性核素^99m^Tc-MDP 2mCi。图像采集条件：excitation= 730 nm，emission= 790 nm；视野直径为12 cm，荧光/X线/放射性核素曝光时间4/1/10 min；照相机设置为4×4像素单元；分别显像后获得荧光、X线和放射性核素融合图像。

##### 1.3.2.3 MRI with sense body采集(1.5 Tesla MRI unit, Philips Healthcare, The Netherlands)

小鼠MRI显像无需注射造影剂，显像序列为：T1WI、T2WI、FLAIR。记录脑内异常信号灶的数量、部位、大小等信息。

#### 1.3.3 病理检测

显像完成后深麻醉处死裸小鼠，在无菌条件下取出异常眼球、完整脑组织，若肉眼能观察到眼、脑组织存在绿色肿块(GFP)，则提示有脑转移病灶并拍照做记录；脑组织和部分病灶组织以10%甲醛溶液固定，行常规石蜡包埋切片，H&E染色，光学显微镜下观察转移情况，另外部分病灶进行体外细胞培养。

#### 1.3.4 体内外筛选肺腺癌脑转移亚株

将脑转移肿瘤组织，剪碎成1 mm-2 mm直径的组织块，放置于培养瓶中行原代细胞培养(培养条件同前)，待细胞贴壁后增殖生长至约90%密度时按1:2比例传代扩增。将此体外扩增脑转移细胞再次经左心室注射接种裸小鼠，重复体内建模-体外培养增殖过程，经4个循环后筛选出具有较高潜能脑转移的肺腺癌细胞亚株。

### 1.4 各轮次细胞与其源细胞CPA-Yang1的比较

#### 1.4.1 细胞倍增时间

各轮次细胞取1×10^4^个细胞/每孔，接种于24孔板中，培养24 h后取3孔细胞计数并计算均值，按公式TD=t^*^[lg2/(lgNt-lgNo)]得到细胞倍增时间^[16]^，在各时间段，倍增时间是不同的，设定取24 h-48 h时间段；也可使用专业计算软件http://www.doubling-time.com。CPA-Yang1细胞倍增时间见参考文献[11]。

#### 1.4.2 转移率计算

综合以下两方面判断肿瘤转移率：①经病理证实的每组裸鼠脑转移率；②经病理证实的每组脑转移裸鼠的转移部位数量。

## 2 结果

### 2.1 荷瘤小鼠的表现

约在左心室造模6周-7周后可发现模型鼠或出现嗜睡，或1只/2只眼睛失明，或头骨鼓出，这些都提示有脑转移存在，需密切关注小鼠的生命体征，防止过度衰竭而亡(图 2)。

**2:**
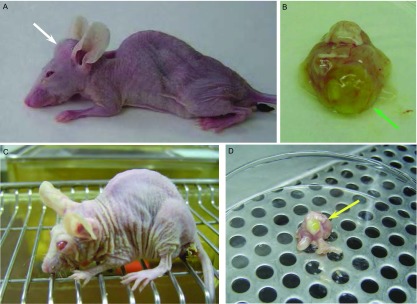
荷CPA-Yang1 GFP细胞左心室造模5周-7周后脑转移裸鼠。A：小鼠自造模第5周始嗜睡，活动日渐减少，进食也下降，白色箭头指向该小鼠额头明显鼓出；B：是A鼠解剖取出的脑组织，绿色箭头所指部分疑嗅脑转移灶；C：小鼠自造模第6周始，眼睛失明，进食全靠触须和嗅觉；D：第7周解剖模型鼠取脑组织，发现绿色（GFP）转移灶（黄色箭头）。 The brain metastasis mice with CPA-Yang1 GFP cells by intracardiac inoculation after 5-7 weeks. A: Since fifth week, the mouse appeared lethargy, activity decreased, eating also fell, the white arrow indicated to the bulging forehead suspected brain metastasis; B: Autopsy of (A) mouse brain tissue, the green arrow indicated bigger lesion of olfactory bulb brain metastases; C: The mouse eyes blind gradually after the inoculation six weeks, eat all by tentacles and smell; D: Dissect out brain tissue in the mouse at seventh week; the yellow arrow indicated the small GFP lesion.

### 2.2 模型鼠分子影像检测

^18^F-FDG micro PET/CT显像，采集的图像由计算机处理后分别获得CT、PET和两者融合的图像(图 3)，能发现脑部浓聚^18^F-FDG，但不能肯定是骨转移还是脑转移。小动物"三合一"活体成像系统在检出腰椎转移的同时发现颈椎以上脑部也有绿色荧光显示病灶，提示可能伴发脑转移(图 4)。带小动物线圈的MRI显像检测荷瘤小鼠的脑内异常信号病灶最为清晰(图 5)。

**3:**
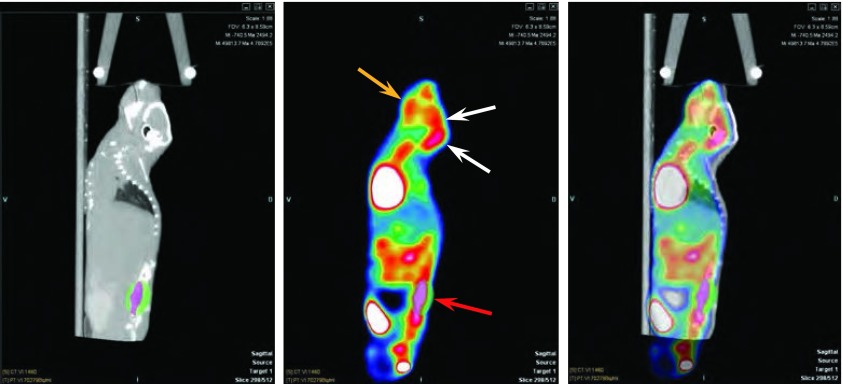
荷CPA-Yang1裸鼠50天行^18^F-FDG micro PET/CT显像（矢状位），从左-右分别为CT、FDG和融合图像。该裸鼠被检出在下颌骨（桔黄色箭头）、腰椎（红色箭头）骨转移，同时伴有后脑部浓聚FDG（白色箭头），但是骨/脑转移无法辨别。 ^18^F-FDG micro PET/CT imagings detected the model mouse after inoculation 50 days (sagittal). CT, FDG and fusion image from left to right respectively. The mouse was found bone metastasis in mandibular (orange arrow) and lumbar (red arrow), but not sure bone/brain metastases as back of the head accumulated FDG (white arrow).

**4:**
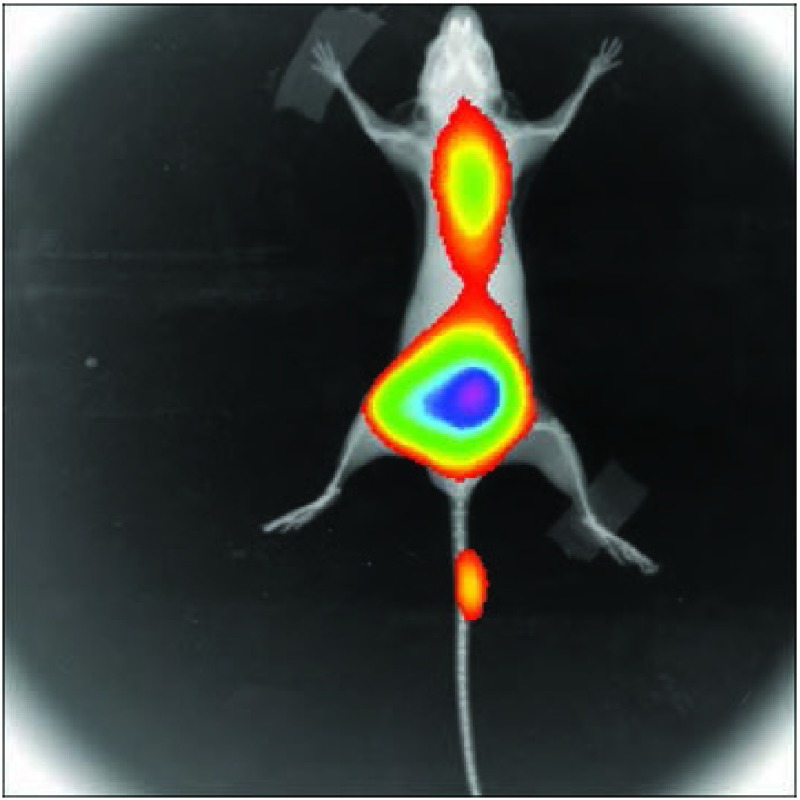
三合一小动物活体成像系统疑该小鼠在胸椎转移的同时伴有脑转移可能。 Small animal *in vivo* imaging system for fluorescence, radionuclide and X ray fused imaging suspected metastasis in thoracic vertebra associated with brain of the mouse.

**5:**
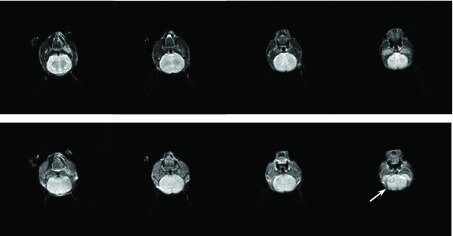
对CPA-Yang1细胞造模40天的裸鼠行带小动物线圈的MRI显像，结果清晰显示小鼠小脑半球转移（白色箭头示）。 MRI imaging of the mouse bearing CPA-Yang1 with small animals coil (sense body) resulted clear display cerebellar hemisphere lesion (white arrow).

### 2.3 细胞生物学变化

#### 2.3.1 细胞倍增时间

每轮次细胞倍增最快时间在24 h-48 h之间，1^st^、2^nd^、3^rd^、4^th^的倍增时间分别为：22.59 h、19.68 h、17.34 h和16.88 h。

#### 2.3.2 体内外筛选脑转移细胞的形态变化

各细胞形态见图 6；早期(p3)及传代半年时间(p34)的CPA-Yang1是人肺腺癌细胞株，分别见图 6A(a)和(b)。细胞呈多核，半贴壁、半悬浮状。经细胞体内外4次筛选，细胞呈悬浮、圆形状变化。

**6:**
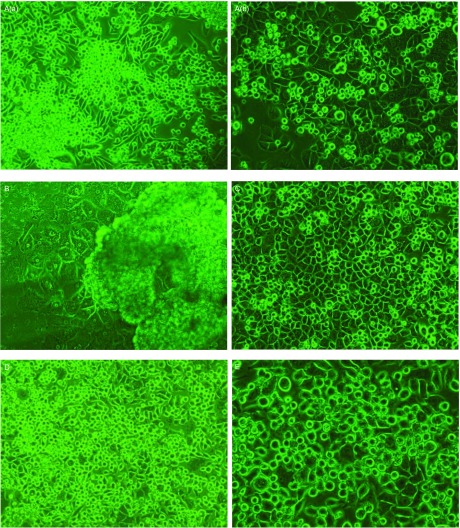
CPA-Yang1细胞的相差倒置显微镜拍摄图片。A：人肺腺癌细胞株（×100），（a）早期细胞形态（p3），多悬浮；（b）传代至（p34），多核；B：模型鼠首代脑转移细胞爬出的状况（×200）悬浮，成团细胞较多；C：第2轮次脑转移细胞，梭形细胞减少，圆形（悬浮）、多边形细胞占多数（×100）；D：第3轮次细胞圆形占多数（悬浮），更活跃（×100）；E：第4轮次悬浮细胞还是占据多数，同时梭形、多边形也有一定比例（×200）。随着轮次的变化，细胞呈小型圆形和悬浮状变化。 Morphology of the CPA-Yang1 cells under the contrast phase inverted microscope. A: Human lung adenocarcinoma cell line (×100), (a) third passage cells more suspension, (b) 34^th^ passage cells suspension and more nucleus; B: First brain metastasis clone presented appearance of growth (×200); C: The brain metastasis clone of 2^nd^ cycle, suspension, polygon cells in the majority; D: The clone of 3^rd^ cycle, suspension cells in the majority (×200); E: Suspended cells of 4^th^ cycle in the majority too (×200).

### 2.4 病理

CPA-Yang1脑转移小鼠病理见图 7，同时可见转移灶的周围血管密度随轮次上升而增加。

**7:**
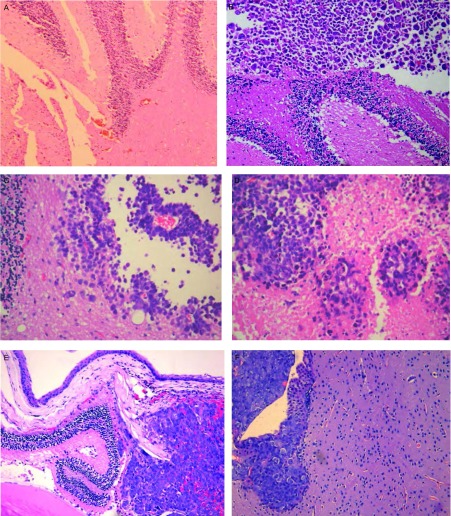
小鼠病理(HE, ×100)。A：正常脑组织；B、C、D：分别是第1、2、3轮次脑转移灶，病灶血供随轮次增加而增加；E：是第4轮次脑-眼转移灶，左下角粉红色为眼晶体；F：第4轮次脑转移灶，细胞呈圆形分布。 Histological features (HE, ×100). A: The brain of disease-free nude mouse; B, C, D is the lesions of brain metastases in 1^st^, 2^nd^, 3^rd^ cycle respectively and diffusive blood supply increases with the cycles; E: The brain metastasis to eye, the lower left corner that pink color is an eye crystal; F: The brain metastasis lesion of 4^th^ cycle, the cells presented circular distribution.

### 2.5 肺腺癌细胞各轮次左心室接种后脑转移发生率

脑转移细胞通过眼管侵袭视神经使得眼球转移-失明，发生率约为6%(2/33)。脑转移发生率与轮次相关(表 1)。根据病理和解剖，每只脑转移小鼠仅发现脑转移灶1个；脑转移病灶大小1 mm^2^-8 mm^2^，有占据整个小脑的，也有位于大脑近嗅脑处的病灶。病灶分别分布在嗅脑2个，大脑4个，小脑3个，视神经2个。

**1:** 脑转移发生率 The morbidity of brain metastasis

Cycle (No.)	Nude mice (*n*)	Intracardiac inoculation in live mice (*n*)	Brain metastasis mice (*n*)	Brain metastasis rate
1^st^	10	9	2	22%
2^nd^	10	8	2	25%
3^rd^	10	8	3	37.5%
4^th^	10	8	4	50%

## 3 讨论

研究肺癌脑转移需要建立可靠的细胞及动物模型，是探讨肺癌脑转移机制和阻断研究的前提。国内外有关肿瘤脑转移模型研究的报道很多，而对中国人肺腺癌患者转移发生率同样较高的脑转移细胞和动物模型的研究却少见报道。

不同的恶性肿瘤细胞具有不同的生物学特性，只有部分恶性肿瘤能产生脑转移，肿瘤转移模型的建立依赖于高致瘤能力的肿瘤细胞，目前研究结果提示能够引起肿瘤发生、转移的细胞只占肿瘤细胞中的少量，而大部分的肿瘤细胞并不具备转移侵袭能力。即使对于同一恶性肿瘤而言，虽然大多是单克隆细胞起源，为同基因型，但其增殖、侵袭和转移特性并不完全相同。肿瘤细胞在转化、生长过程中的自然筛选及其与机体免疫系统的相互作用，使不同肿瘤细胞亚群获得了不同的生物学特性，从而使肿瘤细胞具备了生物学活性的异基因型^[17]^。在可以产生脑转移的恶性肿瘤中，不是所有的肿瘤细胞都具有产生脑转移的能力，只有特定的对脑组织具有特异性亲和力的亚群才具有这种能力^[18]^。血脑屏障能保护患者避免肿瘤细胞轻易扩散至脑组织，反之，能转移到脑的肿瘤细胞必定具有非凡的侵袭潜能。1889年Paget提出的"种子与土壤"假设^[19]^，现代"种子与土壤"学说包括以下内容：①肿瘤是生物学的异基因型，包含不同血管化、侵袭及转移活性的亚群；②转移的整个过程就是特异性转移的肿瘤细胞的筛选过程；③转移的产生依赖于器官微环境与肿瘤细胞多方面的相互作用^[20-22]^。因此，肿瘤细胞株经反复多代接种免疫缺陷型动物并行筛选后，此类亚群细胞得到有效的富集，靶组织转移率渐进升高。一些研究^[23, 24]^报道通过手术肺叶原位嫁接，然后产生脑转移及骨、肾上腺的转移，这个方法无特异性且非常复杂，限制了其在脑转移机制研究中的应用。颈动脉注射肿瘤细胞造模方法^[25]^，由于一次性强制进入脑组织的肿瘤细胞太多导致模型鼠生存期较短，不利于药物疗效观察和向临床医学转换研究，且不符合脑转移生物学特性。左心室注射法很好地模拟了肺癌的血道转移，即通过肺毛细血管床的肿瘤细胞及从形成的微转移灶脱离的肿瘤细胞进入左心室，随血流流向脑部，才能产生脑转移。左心室注射可以减少肺毛细血管床滞留并生长的肿瘤细胞数，增加穿过脑实质的肿瘤细胞数，并延迟因肺癌而致的死亡，延长肿瘤细胞在脑内增殖生长的时间，从而获得更高的脑转移形成的成功率^[26-28]^；我们所选用的造模细胞是自主筛选培养的人肺腺癌细胞CPA-Yang1。以往的裸鼠实验证实其首代致瘤，且尾静脉、左心室种植后均产生溶骨性转移，除了肺部约有10%结节外均未发现其它脏器转移。我们已通过这种方法构建了特异性骨转移的细胞及其动物模型^[11, 29]^，同样很多学者也支持上述理论^[15, 30]^。

因小动物脑转移的检测技术存在局限性，很难通过活体早期影像检测到脑转移灶。这是将CPA-Yang1转染GFP的原因，一则可以借助荧光活体成像系统；二则在解剖取材时可以借以辨别是否转移、转移灶在何处。既往实验中的^18^F-FDG micro PET/CT显像、X线、放射性核素、荧光(三合一)活体成像系统显像均未明确检测出小鼠脑转移灶。分析原因可能是我们使用的放射性核素示踪剂不是难以通过BBB，就是使脑内的本底较高，如^18^F-FDG，造成脑病灶检出灵敏度降低；其次受到设备分辨率的限制，对于小病灶的检出有一定难度；期待将来能开发出灵敏度、特异性更高的示踪剂。荧光显像受细胞内GFP的表达水平及组织衰减影响较大，且我们使用的三合一活体成像系统为平面显像，没有断层或三维重建，对于深部组织的病变难以定位，无法鉴别脑转移和颅骨转移。实验中偶然发现小动物线圈MRI扫描能清晰发现脑转移灶并解剖根据(GFP)捕捉病灶。于是，在以后的检测裸鼠脑转移时，行MRI(with sense body)扫描+解剖查看绿色病灶(GFP)，明显提高了脑转移灶的检出率，尤其是深部、较小脑组织转移灶。但是由于分辨率等的设备技术问题，早期微小转移灶的检出还存在一定的难度，寄希望于更灵敏的micro MRI。

CPA-Yang1-BR人肺腺癌脑转移细胞株及其动物模型的成功建立为进一步筛选靶向脑转移细胞奠定了基础，也为系统研究肺癌的复发、转移和治疗提供了一个良好的实验平台，并将在今后的肺癌分子生物学的研究中发挥更大的作用。同时，小动物线圈MRI或micro MRI活体显像是检测小鼠肺癌脑转移敏感、准确、无创的显像方法。
